# Dogs at school: a quantitative analysis of parental perceptions of canine-assisted activities in schools mediated by child anxiety score and use case

**DOI:** 10.1186/s40723-022-00097-x

**Published:** 2022-03-05

**Authors:** Wendy Irene Fynn, Jessica Runacres

**Affiliations:** University of Derby, Enterprise Centre, Bridge Street, Derby, DE1 3LA UK

**Keywords:** Animal-assisted activities, Canine-assisted activities, Dog therapy, Schools, Acceptability

## Abstract

Canine-assisted activities in schools can benefit students’ educational, emotional, and social needs. Furthermore, they could be an effective form of non-clinical mental health treatment for children and adolescents. In the United Kingdom, school dogs are growing in popularity, however, little is known about how parents perceive canine-assisted activities as a treatment option. This is important as parental perceptions can influence engagement, whilst lack of awareness can become a barrier to treatment. This study uses a cross-sectional design to quantitatively explore the acceptability of canine-assisted activities amongst UK-based parents (*n* = 318) of children aged six to 16 (*M* = 10.12, SD = 3.22). An online survey used a treatment evaluation to determine acceptability across three use-cases. These included a child reading to dogs to improve literacy skills, a child interacting one-to-one to foster greater self-esteem and social skills, and a classroom dog to improve student behaviour and motivation. Additionally, the scale for generalised anxiety disorder was used to rank child anxiety as high or low, where high was a score equal to or above the UK clinical borderline threshold. The results found canine-assisted activities were less acceptable for the behavioural than the reading and social use-cases. Furthermore, parents of children with high anxiety had higher acceptability scores than parents of children with low anxiety for the reading and social use-cases but not for the behavioural use case. These findings suggest that UK parents' acceptability of canine-assisted activities in schools is mediated by child anxiety score. Furthermore, that parents may be less aware of the benefits of classroom dogs than other types of school-based canine-assisted activities.

## Introduction

The World Health Organization (WHO) states that 13% of children and adolescents aged ten to nineteen experience mental health issues (WHO 2021). The UK statistic is similar, with 12.8% of those aged five to nineteen diagnosed with a mental health disorder in 2017 (Sadler et al., 2018). Anxiety-related emotional disorders were the most prevalent amongst those diagnosed including generalised anxiety disorder (GAD), which is persistent anxiety unrelated to a specific circumstance (Sadler et al., 2018). The National Institute for Health and Care Excellence (NICE) guideline for managing anxiety amongst children and young people lists treatment options such as counselling, cognitive behavioural therapy, and medication (NICE, 2014). However, accessing psychological therapy through Child and Adolescent Mental Health Services (CAMHS) has become increasingly difficult due to resourcing issues (Anderson et al., 2017; Sharpe et al., 2016; Sin et al., 2010) and growing demand (NHS, 2019a; Thorley, 2016). This has only become more pronounced since the start of the COVID-19 pandemic (Robinson, 2021).

Although slightly different in their overall approach, both animal-assisted activities (AAA) and animal-assisted therapies (AAT) have been found to be effective in managing anxiety and depression in both children and adults (Molnár et al., 2020; Murray et al., 2019; Waite et al., 2018; Wilson et al., 2017). As such, complementary therapies such as AAT could be viable for use alongside traditional pharmacological and talking treatments. Studies which use animals in psychotherapeutic settings have had positive outcomes when treating adolescents with anxiety and depression (Wilson et al., 2017). Furthermore, some studies have found that therapy involving animals is more acceptable than medication when treating children with externalising behaviour problems (Dravsnik et al., 2018; Rabbitt et al., 2014).

The fields of AAA and AAT are similar in their inclusion of an animal as a core component of the process. However, AAT involves a trained health professional working towards a measurable goal (Friesen, 2010; Maujean et al., 2015), whereas AAA is a more informal, less structured approach (Maujean et al., 2015). Both fall under the term animal-assisted interventions (AAI) (Pet Partners, n.d.). AAI and canine-assisted activities (CAA) have been found to be beneficial for treating mental health issues (Jones et al., 2019; Maber-Aleksandrowicz et al., 2016). This is especially pertinent in the UK given the challenges surrounding CAMHS access (Anderson et al., 2017; Thorley, 2016). One London-based organisation uses CAA to improve communication, social skills, and reduce anxiety amongst adolescents with special educational needs (SEN) (Waggy Tails Club, n.d.). Referrals can be made via CAMHS and the activities are run entirely by a team of volunteers and their dogs (Petley, 2019). Although no research has been conducted to assess the impact of this particular intervention, both AAI and CAA have been found to improve psychosocial outcomes amongst children and young people with intellectual disabilities (Maber-Aleksandrowicz et al., 2016) and Autism Spectrum Disorder (ASD) (O’Haire, 2017).

CAA is particularly relevant given that contact with animals is an important and unavoidable aspect of our society (DeMello, 2012). Uses have burgeoned in recent years from support dogs on planes (Hauser, 2020) to CAA in hospitals (Hinic et al., 2019; Perez et al., 2019). This demand may have been influenced by the success of reading programmes such as the USA’s CARE to Read, and Pets as Therapy’s Read2Dogs in the UK. Both are backed by wide literature supporting the benefits of children reading aloud to dogs (Hall et al., 2016; Henderson et al., 2020; Kirnan et al., 2015; Noble & Holt, 2018; Rousseau & Tardif-Williams, 2019). This includes increased on-task behaviour (Bassette & Taber-Doughty, 2013) and improvement in measurable reading skills such as fluency, accuracy, and intonation (Barber & Proops, 2019). Dogs are said to elicit these outcomes by influencing behaviours which in turn impact reading, including improving confidence and reducing stress and anxiety (Hall et al., 2016; Henderson et al., 2020). As such, an emerging body of research is now exploring the wider mental health benefits of CAA for typically developing children and students (Brelsford et al., 2017; Harris & Binfet, 2021; Kropp & Shupp, 2017; Sin et al., 2010).

Classroom dogs appear frequently in CAA research. Here, a well-trained dog is present in the classroom during lessons, either confined to a bed/crate or allowed to roam during teaching (Anderson & Olson, 2006; Hergovich et al., 2002). Introducing a dog to the classroom during lessons has been found to improve pupil behaviour and socialisation (Kurt & Ortbauer, 2003), and foster a more positive learning environment (Beetz, 2013; Bradley & Maldonado, 2013; Brelsford et al*.*, 2017) provided any medical and cultural barriers are taken into consideration. A recent UK-based study used mixed-methods to explore the benefits of classroom dogs (Mercer, 2019). Interviews were conducted with one staff member from three schools, and a survey was completed by ten respondents. Identified themes related to emotional, behavioural, and educational benefits (Mercer, 2019). Educational benefits included already-established advantages of including dogs in reading activities (Kirnan et al., 2015; Le Roux et al., 2014), as well as during teaching and to motivate pupils. Furthermore, each of the interviewees cited an example of the dog calming a pupil as evidence of the emotional benefits of CAA (Mercer, 2019). Whilst the sample size is small, other qualitative studies have had similar findings (Daly & Suggs, 2010; Hergovich et al., 2002; Kortschal & Ortbauer, 2003; Noble & Holt, 2018).

Classroom dogs can also support students with SEN such as Emotional Behavioural Disorder (EBD) gain skills in responsibility, empathy, and respect (Anderson & Olson, 2006). In one ASD classroom, a pre–post study found that the presence of a dog significantly improved social functioning and enthusiasm for school attendance (O’Haire et al., 2014). Follow-up measures were not taken, therefore the extent to which the results were due to the novelty of the dog is unknown. In some multicultural classrooms dogs can contribute to better social integration by enabling emotional expression and fostering positive relationships between students and teachers (Correale et al., 2017). Whilst the literature to-date does suggest that dogs have benefits for the mental health of children and adolescents via human–animal interaction, further quantitative research using larger sample sizes is required.

There is growing support in the UK for the inclusion of CAA in mainstream schools. Sir Anthony Seldon and Education Secretary Damian Hinds have both publicly stated their support for bringing more dogs into classrooms (Coughlan, 2019). However, little is known about how parents perceive the intended benefits of this (O’Reilly et al., 2018). Whilst CAA has been shown to benefit mental health in schools, parental buy-in needs to be considered. Parental perceptions of treatment have been shown to influence motivation and engagement in the treatment or therapy itself (Hackworth et al., 2018; Mendez et al., 2009; Nock & Photos, 2006). Furthermore, low acceptability and lack of awareness of services are key barriers to treatment uptake (Anderson et al., 2017; Rabbitt et al., 2014).

A relationship between parental perceptions and treatment efficacy has been found in paediatric healthcare, where factors including attitude, perceived behaviour control, and perceived norm predicted behaviours related to both obesity monitoring (Andrews et al., 2010) and gluten-free diet (Marsden et al., 2019). In both cases, a positive parental attitude was found to significantly contribute towards behaviour, namely actively undertaking the health behaviour being studied. Therefore, higher acceptability of CAA might increase the likelihood of parents utilising such options for their child.

Two separate studies have found parental perceptions of CAA to be positive, ranking dog-assisted psychotherapy as preferable to medication (Dravsnik et al., 2018; Rabbitt et al., 2014). However, these studies did not examine CAA in schools, nor differentiate between various CAA uses. Similarly, two studies published in 2019 explored parental perceptions of CAA using qualitative methods (Harwood et al., 2019; Ward et al., 2019). Key findings from both studies included themes of companionship and emotional well-being (Harwood et al., 2019; Ward et al., 2019). However, they too did not examine CAA in schools or differentiate between uses. To the researchers’ knowledge, no studies quantitatively explore parental acceptability of CAA in schools specifically. This knowledge is an important contribution to the literature given how little is known about public views of animal-assisted interventions (Rabbitt et al., 2014).

The present study seeks to address this gap by exploring parental perceptions of CAA in schools. Acceptability of CAA is quantitatively explored alongside the current mental well-being of the participant’s child, as assessed by a score measuring GAD. The aim is to explore whether parents of children with high anxiety will have a more positive perception of CAA, given their child could potentially benefit from this therapy or activity. Furthermore, variations in acceptability will also be explored by presenting three common use-cases of CAA in schools, namely reading to dogs, classroom dogs, and one-to-one interaction with a dog. This study was guided by the following research questions:

Q1: Do perceptions of canine-assisted activities differ across use-cases?

Q2: Do parents whose children might benefit most perceive canine-assisted activities more positively?

The following hypotheses were tested:

H1: Acceptability will differ significantly across use-cases.

H10: There will be no significant differences in acceptability across use-cases.

H2: Parents of children with high anxiety will have a significantly higher acceptability.

H20: There will be no difference in acceptability across high and low anxiety groups.

## Methods

### Design

This study adopted quantitative methods to explore parental acceptability of CAA. An online survey collected data on the demographics of participants. Three different examples of school-based CAA were presented as vignettes, namely reading to dogs, having a dog present in the classroom, and one-to-one interaction with a dog.

### Participants

Participant requirements were: (1) parents or guardians aged 18 or over, (2) who reside in the UK, (3) with a child aged between 6 and 16 years. Exclusion criteria were those whose child has a mental age of less than 18 months (given via self-report). Prior to data collection, calculations on G*Power suggested a total sample size of 76 in order to have a power of 0.8 and effect size of 0.25.

Recruitment took place for 3 months via social media (Facebook and Whatsapp), a research recruitment website, and running paid ads on Facebook. The final sample consisted of 318 participants with 10 male, 307 female, and one other/non-binary. Corresponding child ages covered the full inclusion criteria of six to 16 (*M* = 10.12, SD = 3.22) (see Appendix 1 for participant demographic data).

### Materials

Qualtrics was used to host the survey. The survey included a scale to measure child anxiety via GAD score, a scale to measure the acceptability of three CAA vignettes, and demographic questions. The latter included attitude towards dogs, parent gender, single-parent status, pet ownership, and number of children in the household. Attitude towards dogs was measured separately for parent and child using two statements answered on a five-point Likert scale, “I am afraid of dogs” and “I avoid interacting with dogs for cultural, religious, or personal reasons”.

Two versions of the survey were created, one with the GAD scale before the vignettes (Child First), and one using the reverse order (Dog First). They were otherwise identical, and data collection occurred simultaneously for both by randomising which survey link participants received. Scales used included the RCADS-P (Revised Child Anxiety and Depression Scale (Parental Version)) GAD sub-scale (Chorpita et al., 2000) and the TEI-SF (Treatment Evaluation Inventory (Short Form)) (Kelley et al., 1989). Permission to use both was obtained.

The TEI-SF was used to measure the acceptability of three vignettes, each presenting a form of CAA in schools (see Appendix 2). Each vignette included an explanation of the child’s problem behaviour, a description of the CAA used, and the intended outcome. Each vignette was based upon literature supporting the use of CAA for the given issue; however, they were not piloted with teachers or parents. The reading vignette is rooted in studies which have found that reading to dogs statistically significantly improves measurable reading outcomes (Barber & Proops, 2019; Kirnan et al., 2018; Le Roux et al., 2014; Rousseau & Tardif-Williams, 2019). The behaviour vignette drew upon literature which supports the potential for a classroom dog to reduce externalising behaviour amongst students and support social cohesion and learning (Beetz, 2013; Brelsford et al., 2017; Kortschal & Ortbauer, 2003). The social vignette drew from studies which suggest that one-to-one interaction with a therapy dog can reduce anxiety and stress and improve social skills in children (Beetz et al., 2012; Esteves & Stokes, 2008; Jones et al., 2019).

### Procedure

Ethical approval was obtained prior to commencing the study. An invitation to participate, which contained a link for each survey, was sent out on social media, shared with university groups, parent-related pages, and forums, using the researcher’s Facebook profile. The study was also listed on Callforparticipants.com.

The first survey page included information on the study’s aims alongside a photograph of the researcher and their therapy dog. The next pages explained the participant’s rights, provided data protection information, and gave a consent form. Participants were required to create an anonymous participant ID before proceeding. In both surveys, the first questions collected demographic data on the participant’s household. In one survey, instructions on how to complete the TEI-SF questions appeared before the three vignettes. The RCADS-P GAD sub-scale was then given before the final set of child demographic questions. In the other survey, the RCADS-P GAD sub-scale was presented directly after the household questions, followed by the child demographics, the TEI-SF instructions, and finally the vignettes. The ordering of the three vignettes was randomised in both surveys.

Debrief and final consent concluded both surveys. Participants could withdraw from the study up to 14 days after completing the survey. Parents were permitted to complete the study multiple times if they had more than one eligible child.

### Analysis

Before commencing the analysis, reliability was verified using Cronbach’s Alpha (RCADS-P GAD sub-scale = 0.901 and TEI-SF = 0.906). GAD total score was classed as either high or low using NHS clinical thresholds adjusted for age and gender differences (Wolpert, 2012). Scores that were borderline or above were classed as high for the purposes of the study. These thresholds are only appropriate for ages eight and above, hence analyses using the GAD score did not include data for participants whose children were aged six or seven.

Initial statistical analyses ensured that the ordering of the questions did not impact the survey results. Namely, that asking the child-related questions before or after the vignettes had no significant effect on either GAD or TEI-SF score.

## Results

In total, 549 people commenced the study and 347 completed it. However, 29 participants were located outside the UK and so were excluded. The final sample therefore consisted of 318 participants. An independent T-test checked the impact of question order (Dog First vs Child First) on GAD score. There were no statistically significant differences (*t*(316) = 0.48, *p* = 0.64). A Mann–Whitney test checked the impact of question order on TEI-SF score across the three vignettes. Question order did not have a statistically significant impact on the vignettes, Reading (*U* = 12,384, *z* = 0.28, *p* = 0.78), Behaviour (*U* = 12,316, *z* = 0.19, *p* = 0.85), or Social (*U* = 13,713.5, *z* = 1.94, *p* = 0.05).

The first hypothesis was that acceptability would differ significantly across intervention cases. Data from all participants were analysed (*n* = 318). A total acceptability score for each vignette was used for every participant. The assumptions of normality were violated; therefore, the non-parametric Kruskal–Wallis H Test was used for the analysis. Results indicated that median acceptability scores were statistically significantly different between groups, *x*^*2*^(2) = 3.06, *p* < 0.001. Subsequently, pairwise comparisons were performed using Dunn's (1964) procedure with a Bonferroni correction for multiple comparisons. Adjusted *p*-values are presented. The post hoc analysis revealed a statistically significant difference in acceptability score for behaviour (Mdn = 33) and reading (Mdn = 35) (*p* < 0.001), and behaviour and social (Mdn = 35.5) (*p* < 0.001), but not for reading and social (*p* = 0.53) (Table 1).

Finally, the participants were grouped into low, moderate, or high acceptability according to their mean item score for the TEI-SF for each vignette. This was performed using a similar method to a previous study (Rabbitt et al., 2014): a score of 3.00 was classed as moderate, anything between 1.00 and 2.99 as low and anything between 3.01 and 5.00 as high. Over 80% of participants had high acceptability for both reading and social, whereas only 70.75% of participants had high acceptability for behaviour (see Table 2).

**Table 1:** Descriptive statistics for means comparison of vignette using acceptability

Vignette	*n*	*M*	SD	*V*	SW	SW Sig
Reading	318	33.7	8.07	65.12	.93	.000
Social	318	34.92	6.48	41.96	.93	.000
Behaviour	318	31.11	8.05	64.85	.96	.000

*n* represents sample size, *M* is mean, SD is standard deviation, *V* is Variance, SW is Shapiro–Wilk statistic, and SW Sig. is SW is Shapiro–Wilk significance

**Table 2:** Vignette acceptability rating according to mean item score (TEI-SF)

Ratings	Vignette		
	Reading	Social	Behaviour
Low	14.78%	7.55%	25.47%
Moderate	2.20%	2.51%	3.78%
High	83.02%	89.94%	70.75%

The second hypothesis was that parents of children with high anxiety would have higher acceptability. Participants whose children were six or seven years old were excluded from this analysis, therefore this was a sub-set of the full dataset in which *n* = 230. A two-way mixed ANOVA was performed using acceptability as the DV, vignette (reading, social or behaviour) as the within-subjects variable and anxiety (high or low) as the between-subjects variable. A single outlier was found during boxplot inspection, in the High-Social condition. Acceptability was not normally distributed, as assessed by Shapiro–Wilk’s test (*p* < 0.001) and with negative skewing in all six conditions. A LOG10 transformation was used, however the data were still skewed. The ANOVA was run regardless due to its robustness to Type 1 errors (Blanca et al., 2017).

Descriptive statistics for the transformed data indicated a mean difference for GAD-TS between high and low for all three vignettes, with Behaviour-Low combination showing the highest overall score (*M* = 3.72). Greenhouse–Geisser correction was used to interpret the results (Maxwell & Delaney, 2004). There were significant main effects for within and between subjects, and the interaction between group and vignette, *F*(1.88, 427.43) = 5.32, *p* = 0.006, partial η^2^ = 0.02, ε = 0.94 (Table 3).

**Table 3:** Descriptive statistics for acceptability as a function of vignette and child anxiety (transformed data)

Vignette	Group	*n*	M	SD	SW	SW Sig
Reading	Low	140	3.5	1.07	0.99	0.32
	High	90	3.04	1.15	0.97	0.02
Social	Low	140	3.33	0.92	0.98	0.04
	High	90	3.05	0.96	0.97	0.05
Behaviour	Low	140	3.72	0.97	0.99	0.12
	High	90	3.66	1.165	0.98	0.27

*n* represents sample size, *M* is mean, SD is standard deviation, SW is Shapiro–Wilk statistic, and SW Sig. is SW is Shapiro–Wilk significance

Follow-up tests using estimated marginal means (Univariate) were conducted. There was a statistically significant difference between groups for reading (*F*(1, 228) = 9.47, *p* < 0.001, η_p_^2^ = 0.04) and Social (*F*(1, 228) = 5.00, *p* = 0.03, η_p_^2^ = 0.02), however not for behaviour (*F*(1, 228) = 0.16, *p* = 0.69, η_p_^2^ = 0.001). Partial eta-squared showed effect sizes for these differences in the reading and social conditions to be small (Cohen, 1988). Pairwise comparisons showed acceptability to be significantly greater for the high group in the reading (*M* = 0.46, SE = 0.15, *p* = 0.002) and social (*M* = 0.28, SE = 0.13, *p* = 0.03) vignettes but not the behaviour vignette (*M* = 0.06, SE = 0.14, *p* = 0.69).

Finally, a multiple regression was run to assess if participant demographics had a significant effect on acceptability. The assumptions were tested and met, including homoscedasticity, a Durbin–Watson statistic of 2.11, and collinearity. Three outliers with standardised residuals greater than + -3 were present, however only one case exhibited risky leverage (Huber, 1981). There were no Cook’s distance values above 1. The residuals were normally distributed and so the results of the regression were interpreted. Demographics included were pet ownership, education, employment, attitude towards dogs, and gender. The model was statistically significant, *F*(5, 312) = 20.82, *p* < 0.001, adj. *R*^2^ = 0.24. However, only attitude added statistically significantly to the prediction (see Table 4). Namely, acceptability increased by 4.85 for every unit attitude increased with a medium-to-large effect size (*f*^2^ = 0.33) (Cohen, 1988).

**Table 4:** Table to show regression coefficients, standard error and significance of demographics and attitude as predictors of acceptability

Variable	*B*	SE_B_	β	95% confidence interval for *B*		*p*
				Lower	Upper	
Attitude	4.85	0.55	0.47	3.77	5.92	0.000
Gender	− 0.43	4.61	− 0.01	− 9.5	8.63	0.93
Pets	1.27	0.91	0.07	− 0.52	3.07	0.16
Child SEN	− 2.42	2.22	− 0.05	− 6.78	1.95	0.27
Child AAI	0.67	2.27	0.01	− 3.79	5.13	0.77

*B* represents unstandardised regression coefficient, SE_B_ is standard error of the coefficient, β is standardised coefficient, and *p* statistical significance

## Discussion

Parental acceptability (*n* = 318) of CAA was positive overall. Over 83% of participants rated CAA as acceptable for reading, 89.94% for social and 70.75% for behaviour. The first hypothesis was that acceptability would differ significantly across the three intervention cases. The results revealed that whilst parents perceive CAA to be acceptable for reading and social, CAA is viewed as significantly less acceptable for behaviour. The second hypothesis was that parents of children with high anxiety would have a significantly greater acceptability for CAA than those with low anxiety. This hypothesis was supported in the reading and social vignettes, however, not in the behaviour vignette. Finally, attitude towards companion animals positively influenced attitude towards CAA. This supports a study by Crossman and Kazdin (2018) which had a similar result.

The finding that CAA is perceived positively contributes to existing literature on parental perceptions of AAI (Boyd & Le Roux, 2017; Harwood, 2019; Malcolm et al., 2018; Rabbitt et al., 2014). This literature predominantly explores perceptions of equine interventions (Boyd & Le Roux, 2017; Malcolm et al., 2018; Tan & Simmonds, 2018), therefore the present study adds an underrepresented perspective on including dogs. Furthermore, this finding is valuable given the influence of parental perceptions on treatment efficacy (Andrews et al., 2010; Marsden et al., 2019).

The first null hypothesis was rejected as there were significant differences between CAA use. This has implications for the existing literature on CAA in schools. Namely, several studies conducted within both SEN and mainstream schools have found that including a dog in the classroom improves student behaviour, emotional expression, social inclusion, and desire to learn (Beetz, 2013; Brelsford et al., 2017; Kortschal & Ortbauer, 2003). However, the results of the present study imply that, despite this evidence, parents do not perceive classroom dogs to be as beneficial as dogs for reading or working one-to-one with a child.

The second null hypothesis was accepted for one of the three intervention cases. Namely, parents in the high anxiety group did not perceive the behaviour vignette more positively than those in the low group. Several studies have found classroom dogs to benefit students with SEN such as EBD including improving empathy (Anderson & Olson, 2006), social skills (O’Haire et al., 2014) and, crucially, emotional expression (Correale et al., 2017). This finding suggests that parents whose children might benefit the most from a classroom dog might not be aware of potential advantages.

A distinctive and overarching finding of this study is, therefore, the disjuncture between the established benefits of classroom dogs (Beetz, 2013; Bradley & Maldonado, 2013; Berlsford et al., 2017; Mercer, 2019) and UK parents’ acceptability. Bridging this gap should be a focus for schools advocating CAA given the role parental perceptions play in both accessing and engaging in treatment or therapy. This is particularly important in the UK where classroom dogs have been recommended by education leaders (Coughlan, 2019).

The results also suggest that schools and future interventions could work closely with parents whose children have SEN including anxiety disorders. Firstly, by having these parents act as advocates of CAA to parents of typically developing students due to their higher acceptability overall. Secondly, schools could work alongside parents whose children have SEN to make them more aware of the benefits of classroom dogs given the benefits of CAA for children with SEN.

The study has several limitations. Firstly, the results of the post hoc analysis showed that attitude to dogs had a significant effect on acceptability with a medium-to-large effect size (Cohen, 1988). This implies that the results of this study might be biased if the sample is predominantly made up of dog lovers. Secondly, using the RCADS-P meant excluding ages six and seven from the analysis due to clinical thresholds being unavailable. Other limiting aspects of the design could include the question ordering, the wording of the vignettes lacking detail, and the placement of a photograph of the researcher and her dog on the first page of the survey. The latter may have influenced which participants went on to complete the survey by appealing more to dog lovers. Parents with multiple children were allowed to complete the survey more than once which may have skewed the data.

The wording of the behaviour vignette may also have impacted the scores. Reading and social described one-to-one CAA while behaviour implied a longer-term group use of CAA, given studies involving classroom dogs are typically six to ten weeks long (Bassette & Taber-Doughty, 2013; Donaldson, 2016; Le Roux et al., 2014). Therefore, the wording might have impacted scores via participants interpreting it as simply bringing a dog into the classroom once-off rather than a sustained intervention.

There were also some limitations related to confounding variables and, as mentioned, participant bias. Firstly, participants predominantly had a positive perception of dogs which may limit generalisation. Secondly, almost half the sample had a pet dog (*n* = 137). This figure is higher than the reported number of UK households which include a child and a dog (24%) (PFMA, 2019). It is therefore possible that participants with a positive attitude towards dogs were more likely to complete the survey. It would be interesting for future research to explore this by comparing the mean attitude towards dogs found in the present study (*m* = 8.9) with a wider sample to ascertain whether it resembles general UK averages or a cohort of dog lovers.

The findings of this study present further opportunities for future study. Firstly, the exploration of parental perceptions towards CAA in schools using in-depth qualitative methods would be useful to help direct future interventions and, importantly, policies on including dogs in school environments. Secondly, future studies could investigate the acceptability CAA within specific demographic groups, for example within Muslim communities where dog ownership might be less common.

Finally, future studies using a similar design could randomise the order of the survey sections and use a different measurement of GAD so that all age groups could be included in the analysis. It would also be useful to present several variations of the behaviour vignette in order to explore the acceptability of classroom dogs in more depth and ascertain whether they are perceived as less acceptable or if the results are related to aspects of the present study’s design.

## Conclusion

The present study explored parental acceptability of CAA in schools alongside the current mental well-being of the participant’s child. The study explored whether parents of children with high anxiety had a more positive perception of CAA than those with low anxiety. Finally, variations in acceptability were explored by presenting three use-cases of CAA in schools. The findings indicate that while parental perceptions of CAA in schools is positive, there is variation across CAA use. Specifically, CAA for reading or one-to-one interventions are viewed as more acceptable than classroom dogs. Furthermore, parents whose children have high levels of GAD have a more positive perception of CAA for reading and one-to-one interventions but not for classroom dogs.

These findings are particularly relevant in the UK where classroom dogs are increasing in popularity, and over 12% of children and young people are diagnosed with a mental health disorder. CAA has the potential to be an effective way of improving students’ emotional and educational needs, however, low acceptability could prevent parents from engaging with or supporting CAA in schools. Future research could be done to explore why parents perceive classroom dogs as less acceptable than other types of CAA, and to direct programmes and policies aimed at increasing awareness of the benefits of having dogs in the classroom.

## Data Availability

Materials available at https://bit.ly/2GSLjOU. The dataset analysed during the current study is available from the corresponding author on reasonable request.

## Appendices

### Appendix 1: Demographic details for study participants across the split sample

**Table:**

	Sample 1 (Child first) (*n* = 190)				Sample 2 (Dog first) (*n* = 128)			
	*M*	SD	Min	Max	*M*	SD	Min	Max
Parent’s attitude to dogs	8.78	1.98	2	10	9.08	1.65	2	10
Age of child	10.21	3.25	6	16	9.99	3.19	6	16
No. of siblings	1.33	1.25	0	12	1.27	0.94	0	4
Child’s attitude to dogs	8.59	2.20	2	10	8.27	2.28	2	10

		*N*				*N*		
Parent gender								
Male		2				8		
Female		187				120		
Other/non-binary		1				0		
Single-parent household								
Yes		37				23		
No		153				105		
Pet ownership								
Other pets incl dogs		38				30		
Other pets excl dogs		47				41		
Only dog/s		47				22		
No pets at all		58				35		
Child gender								
Female		84				66		
Male		105				61		
Other/non-binary		1				1		
Child diagnosed SEN								
Yes		49				28		
No		141				100		
Child attends an AAI								
Yes		28				15		
No		162				112		
Unsure		0				1		
Child’s school has CAA								
Yes		34				14		
No		148				110		
Unsure		8				4		

*M* represents mean, SD represents standard deviation and *N* represents number
